# Supplementary material for: Newborn chicks show inherited variability in early social predispositions for hen-like stimuli
Source: Sci Rep. 2017 Jan 24;7:40296. doi: 10.1038/srep40296 (PMC5259780; doi:10.1038/srep40296)
Supplement: Supplementary Material 1 [file srep40296-s1.pdf]

Supplementary material

Newborn chicks show inherited variability in early social predispositions for hen-like stimuli

Elisabetta Versace, Ilaria Fracasso, Gabriele Baldan, Antonella Dalle Zotte, Giorgio Vallortigara

During the tests, we observed that chicks from all breeds were able visually explore the test stimuli throughout the experiment, shifting their position in the wheel.
To quantify this behaviour, we measured the number of displacements of the body orientation between quadrants (Figure S1).

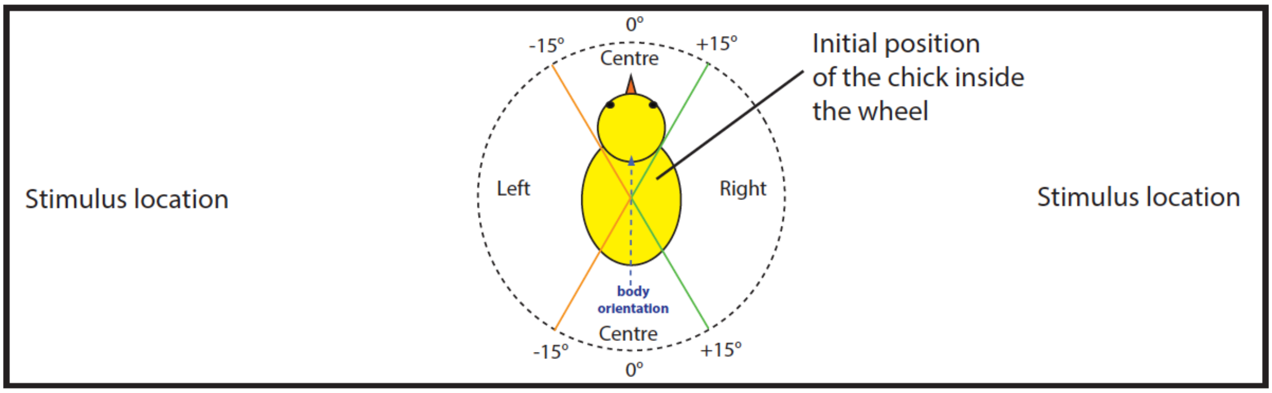

Figure S1.
Initial position of the chick in the wheel inside the apparatus, from the top, and the four quadrants.

The median number of displacements for each breed was respectively: 32 (PD), 61 (PL), 35 (RB), as illustrated in Figure S2. This shows that all breeds were able to move inside the apparatus and visually explore both stimuli.

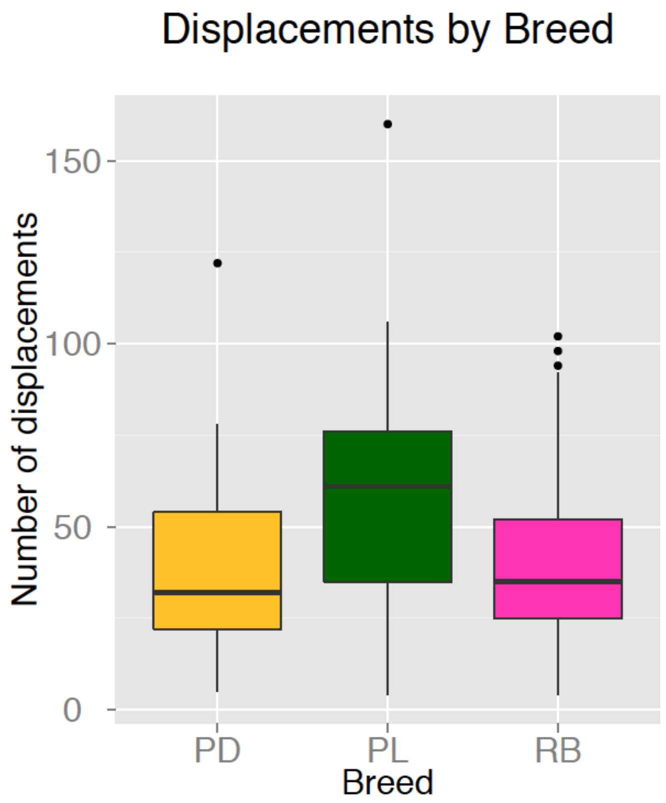

Figure S2
Number of displacements during the test by breed (PD= Padovana, PL= Polverara, RB= Robusta).

The Kruskal-Wallis test shows that breeds significantly differ in the number of displacements (Kruskal-Wallis chi-squared$_2$=10.7456, p=0.0046). Subsequent post hoc tests show that the breed that maintains the preference for the hen-like stimulus throughout the test displaces significantly more than the other breeds: Polverara vs Padovana: W=239, p=0.002; Polverara vs Robusta: W=608, p=0.019; Padovana vs. Robusta: W=367.5, p=0.414.
Thus we conclude that there is no evidence of difficulties in shifting between stimuli that can explain why, after the initial significant preference for the hen-like stimulus observed in all breeds, the Polverara breed is less attracted by the other stimulus.
